# Structural analysis and gasification reactivity of chars derived from the slow pyrolysis of extruded coal fines and recycled plastic

**DOI:** 10.1016/j.heliyon.2024.e39391

**Published:** 2024-10-15

**Authors:** C. Marais, J.R. Bunt, N.T. Leokaoke, H.W.J.P. Neomagus, G.N. Okolo, N.J. Wagner, J.A. Meyer

**Affiliations:** a Centre of Excellence in Carbon-Based Fuels, North-West University, Potchefstroom Campus, Private Bag X6001, Potchefstroom, 2520, South Africa; b DSI-NRF CIMERA, Department of Geology, University of Johannesburg, South Africa

**Keywords:** Coal, LDPE, PP, CO_2_ gasification, BET, Carbon form analysis

## Abstract

The evolution of char resulting from the co-pyrolysis of recycled plastic and discard fine coal, along with the impact of varying plastic additions on the characteristics of the formed char and its subsequent gasification reactivity, remains unexplored. In this study, extrudates were produced using discarded South African Highveld coal fines, combined with either recycled low-density polyethylene (LDPE) or polypropylene (PP), respectively, and charred under a nitrogen atmosphere at three different temperatures (520, 720, and 920 °C). Co-gasification of plastic and coal provides an opportunity to reduce two waste streams simultaneously. The characteristics of the chars produced from the coal fines were compared to those produced from the extrudates formulated at varying plastic content (10, 25 and 50 %wt), using petrographic carbon form analysis, surface area and porosity analysis, as well as XRD carbon crystallite analysis. Thermal fragmentation was observed to degrade the integrity of the extrudates when the plastic content exceeded 10 %, therefore, the chars produced at 920 °C were pulverized (75–150 μm) before undergoing CO_2_ gasification at 800, 825 and 850 °C in the chemical-controlled regime. Carbon crystalline analyses showed that the chars were more ordered with an increase in plastic content. However, even with the more ordered structure, the gasification reactivity increased similarly with an increase in plastic content for both the extrudate derived chars containing LDPE and PP. Petrographic carbon form analysis showed an increase in crack and pore formation with an increase in plastic addition during all stages of the chars’ evolution as the pyrolysis temperature increased. The VRM time factor showed a positive linear correlation with an increase in BET surface area, especially for the LDPE derived chars (R^2^ > 0.7720). Therefore, the observed reactivity increase with an increase in plastic content can be correlated to an increase in surface area. Results allude to the possibility of increasing gasification rates in Industrial gasifiers while reducing both coal and plastic waste. Further work should be conducted to minimise the thermal fragmentation propensity of the plastic-containing fine coal bound extrudates to make the extrudates suitable for fixed bed gasification.

## Highlights

• Chars from extrudates containing plastic showed higher degrees of pore and crack formation compared to chars from coal fines.
• Maximum porosity occurred at a pyrolysis temperature of 720 °C, indicating pore growth mostly occurred during the pyrolysis.
• XRD analysis of carbon crystalline structure indicated that plastic addition lead to the production of more ordered chars.
• CO_2_ gasification reactivity of chars produced from plastic and coal fine extrudates increased an increase in plastic content.
• The VRM time factor has a positive linear relation with an increase in surface area (R^2^ > 0.7720) for chars containing LDPE.

## Graphical abstract

**Figure:**
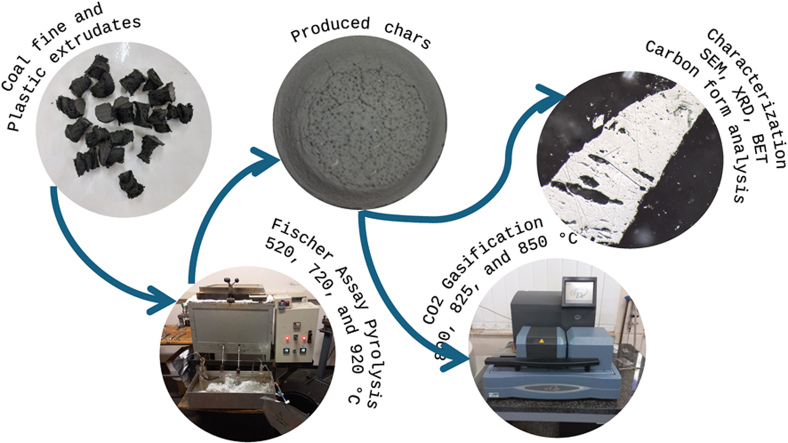

**Table:** 

**Nomenclature**		
**Symbol**	**Description**	**Units**
$A_{0}$	Ash content	*%wt*
$d_{002}$	Interlayer Spacing	*Å*
$E_{d}$	Demineralization efficiency	*%*
$E_{a}$	Activation Energy	*kJ.mol*^*−*^*^1^*
$L_{a}$	Crystallite diameter	*Å*
$L_{c}$	Crystallite height	*Å*
$k_{s0}^{\prime }$	Lumped pre-exponential factor	*min*^*−*^*^1^*
$N_{ave}$	Average stacking number of aromatic layers	*-*
t	reaction real time	*min*
$t_{f}$	time factor	*min*^*−*^*^1^*
QOF	Quality of fit	*%*
X	Conversion	*-*
β	Full Width at Half Maximum (FWHM) values	*°*
θ	peak position, X-ray diffraction angle	*°*
λ	X- ray wavelength	*nm*

## 1 Introduction

The main uses of coal in South Africa are for electricity generation and liquid fuel production, and these two industries account for approximately 90 % of the domestic coal consumption [1]. The Lurgi Fixed Bed Gasification technology is used to produce a variety of hydrocarbons from coal and employs the Fischer-Tropsch process for fuel production [2]. This robust gasification process can be adapted to handle feedstock having a wide variety of properties [2], which is crucial, since there is mounting pressure to investigate the co-gasification of coal together with waste streams, thereby diversifying the energy mix and lowering the country's dependency on fossil fuels [3]. Coal fines are often discarded since its reduced size (<1 mm) increases the difficulty of industrial handling and transportation [4]. Additionally these coal fines are not used in the Sasol lump coal gasification process as it requires particle diameters >6 mm^4^. South Africa discards approximately 60 Mt of coal fines per annum [5], and the use of these discarded coal fines in an agglomerated form within the fixed bed gasifier provides a financial opportunity for use in thermal applications [6]. Research aimed at enhancing the size of coal particles for industrial use, is currently exploring agglomeration processes involving municipal wastes and coal [7].

South Africa has the eleventh most mismanaged plastic waste globally [8]. In a previous study, the extrusion of discarded coal fines from the Highveld Coalfield together with varying concentrations (10, 25, 50, 75, and 100 %wt) of recycled waste LDPE and PP were investigated [9]. It was concluded that the extrudates (10 mm diameter) had mechanical strength and water resistance properties that exceeds the standards for coal agglomerates, while iso-conversional methods determined that the addition of plastic lowered the overall activation energy required during pyrolysis [9]. The Fischer Assay slow pyrolysis products derived from the extrudates were investigated in a subsequent study, finding that the product yields of char, condensable products, water, and gas had little variance from the additive model; however, the product characteristics showed definitive interaction between the coal and plastic [10].

Investigating the co-gasification of extruded waste plastic and discarded coal fines would be beneficial for maximizing the utilization of both waste streams. Coal gasification is a two-step process, including: (1) the devolatilization of the coal during pyrolysis; and (2) the subsequent reaction of the chars with multiple gases to produce syngas [11]. Plastics yield minimal char, and their gasification is complex due to extensive cracking, and due to the abundant hydrocarbon chains (such as waxes and oils) being produced during pyrolysis [10,12]. Since plastics produce almost no char [10], the slow heterogenous gasification of chars is not as prominent during gasification as it is for coal. Given the robust nature of the Lurgi Fixed Bed gasifiers, fuel sources with widely different properties can be blended into the main coal fuel source [2]. Therefore, the rate limiting char gasification step for these mostly coal containing fuel blends used by the Lurgi Fixed Bed Gasifiers is noteworthy. It is crucial to understand the effect that varying plastic content within the extrudates has on the formation and evolution of the chars as the final pyrolysis temperature is increased (step 1 in gasification).

Coal chars have been extensively analysed to better understand their characteristics and their evolution as the pyrolysis temperature increases [[13], [14], [15], [16]]. However, the properties of chars produced from coal and plastic extrudates have not received much attention. Wu et al. [17] documented the char morphology resulting from premixed blends of white polyethylene (WPE) and coal, as well as black polyethylene (BPE) and coal, produced at 550 °C. The study employed a fast-heating rate of 20 °C/s, and microscope images revealed that blends with WPE generated larger pores on the char surface, suggesting an enhanced rapid escape of volatile matter during pyrolysis. Furthermore, the rough surface observed from the coal and BPE char can facilitate the interaction between the aliphatic radicals released from the BPE and the volatiles released on the surface of the coal [17]. Zhang et al. [18] reported on the effect of the placement position of low-rank Chinese coal relative to a 5 % addition of HDPE (i.e. HDPE placed below coal, HDPE placed in between coal, etc.) on the pyrolysis product yields and characteristics when increasing the temperature to 800 °C under an argon atmosphere. Fourier transform infrared spectroscopy illustrated that the intensity of the methylene peaks increased with the addition of HDPE. The X-ray diffraction (XRD) results reported that the introduction of HDPE increased the order of the carbon structure within the char samples, which increased the degree of graphitization [18]. Vivero et al. [19] also identified increases in the intensity of the aliphatic peaks with the addition of plastics to coking coal during slow pyrolysis by using FTIR with diffuse reflectance mode.

Only a few studies have investigated the gasification of plastics with the addition of coal or biomass, and most of these studies focused on evaluating the product distribution and syngas composition [3,12,[20], [21], [22], [23], [24]]. There is currently little available literature on the gasification kinetics of the co-pyrolysis of chars derived from coal and plastic blends. Zhang et al. [25] tested the CO_2_ gasification kinetics of pulverized char derived from a semi-coke and a polyethylene film after it was heat treated. The addition of 20 % polyethylene produced the highest CO_2_ gasification reactivity. The reactivity of all of the blends that contained plastic (10–25 %) was higher than that of the semi-coke alone, and the gasification reactivity could be adequately described by both the random pore model (RPM) and the volume reaction model (VRM) [25]. Sahu et al. [26] also found that the CO_2_ gasification reactivity and the syngas calorific value increased when HDPE was added to the high ash coal from the Jharia coalfield. The char became highly porous when HDPE was added, making it suitable for the Boudouard reaction. During TGA analysis from ambient up to 1000 °C, the gasification stage was best described with diffusional models [26].

Since changes in the structural and chemical properties of the chars produced during the pyrolysis step can affect the final gasification kinetics, this study aims to improve upon the understanding of the evolution of the chars derived during the co-pyrolysis of coal and plastic extrudates. This was done by increasing the final pyrolysis temperature from 520 to 920 °C, a temperature range similar to the pyrolysis temperature range of Lurgi fixed bed gasifiers (300–900 °C) [27,28], and varying plastic concentration. This was achieved by characterizing the produced chars using scanning electron microscope (SEM), surface area and porosity analysis, XRD (carbon crystallite analysis), and petrographic carbon form analyses. Since the addition of plastics during pyrolysis causes a significant increase in liquid product production [10], only the gasification propensity of the pyrolysis chars with a lower plastic addition (0, 10, 25, and 50 %wt) were investigated in this study. Furthermore, the CO_2_ gasification kinetics of the pulverized chars produced at 920 °C were determined and modelled using the VRM. By enhancing the comprehension of the gasification kinetics of chars produced from coal and plastic extrudates, this study aims to contribute to the optimization of the utilization of both waste streams.

## 2 Methods

### 2.1 Samples

**Fig. 1:**
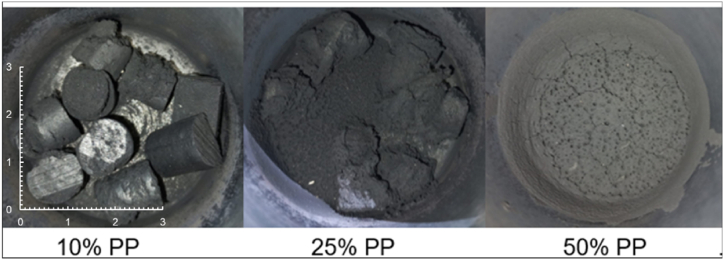
Chars produced using PP containing extrudates at 920 °C.

The chars produced from the slow pyrolysis of extrudates (diameter ±10 mm) derived from discarded fine coal (<1 mm) and recycled plastic were generated within a modified Fischer Assay setup. The extrusion and pyrolysis methods used are described in previous studies [9,10]. Furthermore, in this paper only the chars produced from the raw coal fines and the extrudates containing lower plastic concentrations (10, 25, and 50 % plastic) were selected and reported on for both the extrudates containing LDPE and PP, respectively. The final pyrolysis temperatures at which the chars were produced were 520, 720, and 920 °C. The chars were characterised to gain insight into their evolution with changes in temperature and plastic concentration. Depending on plastic concentration, the chars produced during pyrolysis did not remain in the original form of the extrudates (Fig. 1).

At higher plastic concentrations, char was found evenly spread around the reactor walls, which is a visual sign that swelling occurred during pyrolysis. With the increase in plastic content the swelling compromised the original structure of the extrudates. At 10 % plastic addition, the chars remained in the shape of the extrudates after being exposed to the final pyrolysis temperature. The 25 % plastic containing extrudates started to form a cake; however, the outline of the original shape of the extrudates within the cake can still be observed, whereas the 50 % plastic containing extrudates formed a cake with no trace of the original individual extrudates. Visually, there was no noticeable change in swelling/caking behaviour with variation in the final pyrolysis temperature, or when comparing LDPE and PP addition. This suggests that the change in plastic content is responsible for the caking phenomena. All chars were found to be brittle and easily reduced to fines (even at 10 % plastic addition). However, these chars were maintained in their original structure for all structural analyses performed.

### 2.2 CO_2_ gasification experiments

To ensure gasification took place in the chemical-controlled reaction regime, the chars were crushed and sieved to between 75 μm and 150 μm and iso-thermal gasification temperatures below 900 °C were selected. An SDT Q600 TGA was utilized to gasify a sample of ±15 mg of the char produced during the Fischer Assay pyrolysis of the extrudates at 920 °C. The sample was heated (20 °C/min) under a constant nitrogen flow rate of 100 mL/min to the selected iso-thermal gasification temperature (800, 825, and 850 °C). Since the chars were previously pyrolyzed, insignificant mass loss occurred during heating, only removing any absorbed moisture or remnant volatile matter. The gasification of the chars commenced under a constant CO_2_ flow rate of 100 mL/min at the selected gasification temperature until completion.

### 2.3 CO_2_ gasification kinetics

The gasification kinetic parameters were modelled using the volumetric/homogenous reaction model (VRM) and the random pore model (RPM). These models provided similar results and the VRM model was selected to further discuss the gasification behaviour of the chars. The RPM method and the comparison between the RPM and VRM results can be found in the Supporting Information. When using the volumetric reaction model, it is assumed that the sample contains enough voids that the reactions occur homogeneously throughout the solid phase. The VRM is described by Equation (1) [29].

(1) $$\begin{array}{c} X=1-\exp\left(-t_{f}t\right) \end{array}$$

where $t_{f}$ is the time factor when using VRM and t is the reaction real time.

Arrhenius plots were constructed using Equation (2), and the slope of $\ln\left(t_{f}\right)$ versus $T^{-1}$ was used to determine the activation energy ($E_{a}$) and the intercept was used to estimate the lumped pre-exponential factor ($k_{s0}^{\prime }$) for both models [30].

(2) $$\begin{array}{c} \ln\left(t_{f}\right)=-\frac{E_{a}}{RT}+m\;\ln\left(y_{CO_{2}}\right)+\ln\left(k_{s0}^{\prime }\right) \end{array}$$

The VRM was evaluated using the quality of fit equation (Equation (3)).

(3) $$\begin{array}{c} QOF\left(\%\right)=100\times \left(1-\frac{\sum_{1}^{N}\frac{X_{cal}-X_{\exp}}{X_{\exp}}}{N}\right) \end{array}$$

where the data points used for both models are represented by N. $X_{cal}$ is the calculated conversion and $X_{\exp}$ the experimental conversion [31,32].

### 2.4 Physical-structural properties

#### 2.4.1 SEM

The coal fines, extrudates, and chars were mounted on to pin-type aluminium stubs using double sided adhesive carbon tape. The loose excess particles were blown off using compressed air. The samples were examined using the backscatter mode. The chars and coal samples remained uncoated, whereas the extrudates required carbon coating to enhance conductivity. The images were taken at the North-West University using a Quanta 250 FEGSEM 20 kV at a working distance of 10 mm.

#### 2.4.2 Carbon form analysis

The chars were optically compared to the raw coal fines sample using a petrographic analytical method at the University of Johannesburg. The chars were prepared as polished block mounts using a Struers Tegraforce polisher, following the SANS 7404-2 [33] procedure. A Zeiss Axiolmager M2M reflected light microscope retrofitted with Hilgers Diskus Fossil components and software was used to perform the carbon form analysis, at a magnification of ×500 under oil immersion. A semi-automated point counting stage was used, and the results are reported on a volume percentage (vol%). This petrographic method was previously applied by numerous researchers [34,35], and the categories included in the quantification of carbon form include: coal microlithotype (vitrite, inertinite, carbominerite, bi- and tri-macerals, and rock), devolatilized coal (porous, porous mixed, dense, cracked, and carbominerite), and char form (vitrite pores, porous mixed, dense vitrite, inertite, cracked, oxidised, carbominerite and rock). The nature of the coal fines presented quantification challenges similar to those previously encountered by Bunt et al. [34]. Microphotographs of the samples were taken in reflected white light.

#### 2.4.3 XRD

The XRD analysis was conducted by XRD Consulting and Analytical cc, and the samples (<75 μm) were prepared using a back-loading preparation method. A Malvern Panalytical Series diffractometer fitted with a PIXcel detector along with fixed slits with Fe-filtered Co-Kα radiation was used to produce diffractograms. Different phases were distinguished using X'Pert Highscore plus software alongside PAN-ICSD. The percentage weight (%wt) of each phase amount was approximated using the Rietveld method.

To investigate the carbon crystalline structures of the samples using the gaussian peaks, the mineral matter needed to be removed since the mineral peaks overlap and obstruct the measurements of the gaussian peaks. The samples were demineralized by immersing the samples for 24 h in (1) hydrochloric acid (HCl, 37 %, supplied by Sigma Aldrich), then for 24 h in hydrofluoric acid (HF, 45 %, purchased from Minema chemicals), and again into hydrochloric acid for a final 24 h. The samples and acid mixtures (1:5 sample to acid weight ratio) are continuously agitated in each step. After the final acid wash was completed, the samples were rinsed with water until the pH of the filtrate was 6 or above. The demineralization efficiency (E_d_) was determined using Equation (4) [32].

(4) $$\begin{array}{c} E_{d}=\left(\frac{A_{0}-A_{d}}{A_{0}}\right)\times 100\% \end{array}$$

Where A_0_ is the original ash yield of the samples and A_d_ is the ash yield of the demineralized samples, respectively. The demineralization efficiency was calculated to be between 91.4 % and 97.7 % for all samples. The Braggs and Scherrer equations were used to determine the average carbon crystalline parameters (interlayer spacing (d_002_), crystallite height (L_c_), and crystallite diameter (L_a_)) [36] for the demineralized samples (Equations (5), (6), (7)). The average stacking number of aromatic layers (N_ave_) was calculated using the crystalline parameters and the area below the 00_2_-band and the γ side band (Equation (8)) [32,36]. Where the X-ray wavelength (λ) is equal to 1.789 nm.

(5) $$\begin{array}{c} d_{002}=\frac{\lambda }{2s\text{in}\left(\theta_{002}\right)} \end{array}$$

(6) $$\begin{array}{c} L_{c}=\frac{0.89\lambda }{\beta_{002}\;\cos\left(\theta_{002}\right)} \end{array}$$

(7) $$\begin{array}{c} L_{a}=\frac{1.84\lambda }{\beta_{100}\;\cos\left(\theta_{100}\right)} \end{array}$$

(8) $$\begin{array}{c} N_{ave}=\frac{L_{c}}{d_{002}}+1 \end{array}$$

Additionally, the diffraction angles (θ_002_ and θ_100_) and the Full Width at Half Maximum (FWHM) values (β_002_ and β_100_) were correlated with the peak positions of 002 and 100, respectively.

#### 2.4.4 Skeletal density

The Micromeritics Accupyc II 1340 Gas Pycnometer was used to measure the chars’ skeletal density. The pycnometer uses the outlet pressure of 1.34 bar from a 10 cm^3^ sample container. The sample was placed into the container ensuring that the container is at least 75 % full (±5 g) and then flushed with helium to degas the sample to a pressure of 10 μm Hg. The average of six skeletal density measurements, with a 95 % confidence interval, was reported.

#### 2.4.5 CO_2_ and N_2_-LPGA

A Micrometrics 3-Flex multiport surface area and porosity analyzer was utilized to measure the CO_2_ and N_2_ surface area and porosities of both the produced chars and the extrudates from which the chars originated. Prior to the CO_2_ and N_2_ low-pressure gas adsorption (CO_2_– and N_2_–LPGA) analyses the samples were degassed at 90 °C under vacuum conditions (10 μm Hg) for 12 h. The CO_2_ LPGA analyses were conducted in an ice-water bath, whereas the N_2_ LPGA analysis was conducted using liquid nitrogen at −196 °C [37,38]. The adsorption data was automatically collected by the Micromeritics Flex v6.02 software over a pressure range of 0 < P/P_0_ ≤ 0.033 for CO_2,_ and 0 < P/P_0_ ≤ 0.995 for N_2_ adsorption analysis, respectively. For the surface area determination from N_2_ LPGA analysis, the partial pressure range is limited to 0.30 when analysing coal since this range provides a multi-layer coverage of N_2_ layers which gives a linear plot of 1/[Q(P0/P - 1)] against (P/P_0_) [38]. The N_2_ surface area was evaluated using the BET method; in this case the D-R method was used to determine the micropore surface area. The adsorption pore diameter and volume were determined using the Barrett–Joyner–Halenda (BJH) method. The pore size distribution was calculated with the adsorption isotherm using the BJH method [38].

CO_2_ LPGA surface areas were evaluated at a lower partial pressure range since the micropores are filled at substantially lower partial pressures due to significantly higher saturation vapour pressure of CO_2_ at the analysis temperature of 0 °C^38^. During CO_2_ adsorption, the Dubinin–Radushkevich (D–R) and the Brunauer–Emmett–Teller (BET) isotherm models were used to determine the surface areas of the samples; furthermore, the maximum pore volume and pore width were determined by the Horvath–Kawazoe (HK) method [38]. These calculations were performed using isotherm data with P/P_0_ ≤ 0.033. The N_2_ surface areas of the samples were evaluated using the BET isotherm model. The adsorption pore diameter and volume were determined using the Barrett–Joyner–Halenda (BJH) method. Using the BJH pore size distribution (PSD) data from the N_2_ LPGA adsorption and or desorption isotherm [38], the surface area, pore volume and porosity of the samples can be categorised into their micro-, meso-, and macropore contributions [[39], [40], [41]].

## 3 Results and discussion

### 3.1 Gasification

**Fig. 2:**
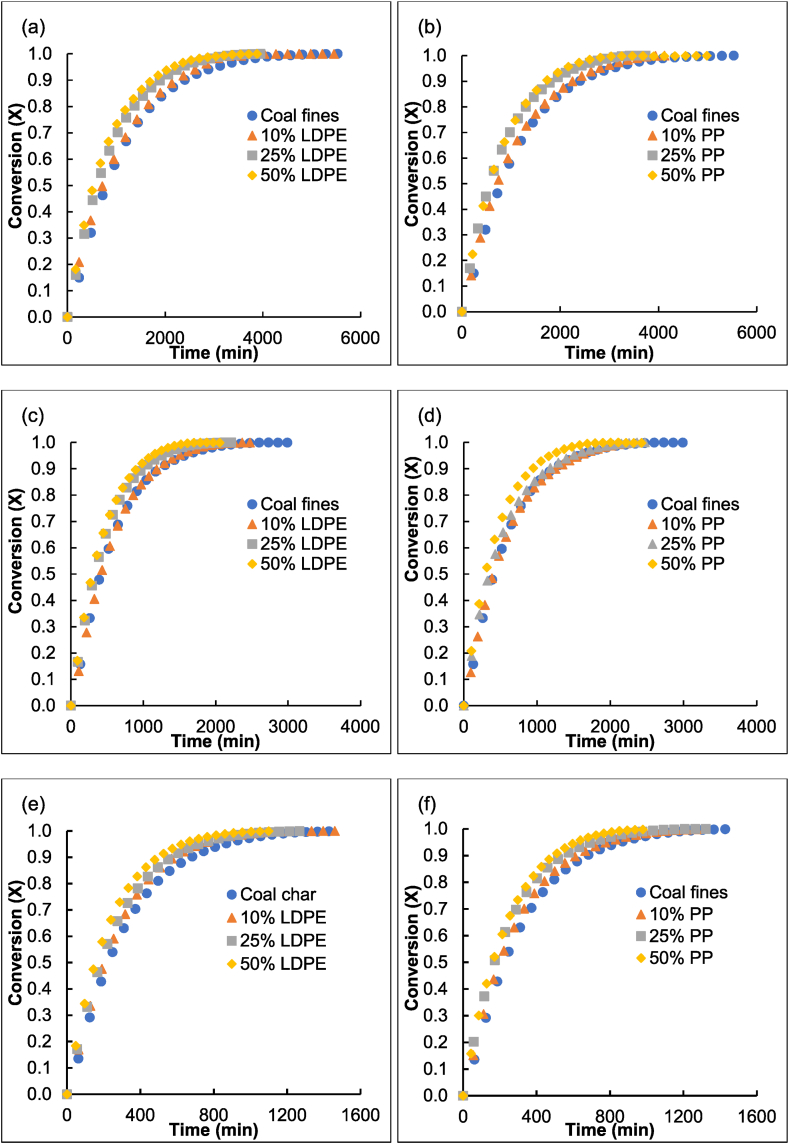
CO_2_ gasification conversion of the chars produced from LDPE containing extrudates at (a) 800 °C, (c) 825 °C, and (e) 850 °C and the chars produced from PP containing extrudates (b) 800 °C, (d) 825 °C, and (f) 850 °C.

The CO_2_ gasification conversion results obtained for the chars can be seen in Fig. 2.

It can be observed that, at every temperature, the gasification rate of the chars consistently increases with an increase in plastic content for both the chars derived from the LDPE and PP containing extrudates. Both Zhang et al. [25] and Sahu et al. [26] found that the gasification reactivity of the char increased when plastic was added to a coal source. Zhang et al. [25] also found that the reactivity of the chars increased with an increase in plastic content in the original blend up to 20 % plastic addition, however, at 25 % addition the reactivity no longer increased. In this study, the reactivities of chars produced from extrudates containing both LDPE and PP increased as the plastic content within the extrudates increased.

**Fig. 3:**
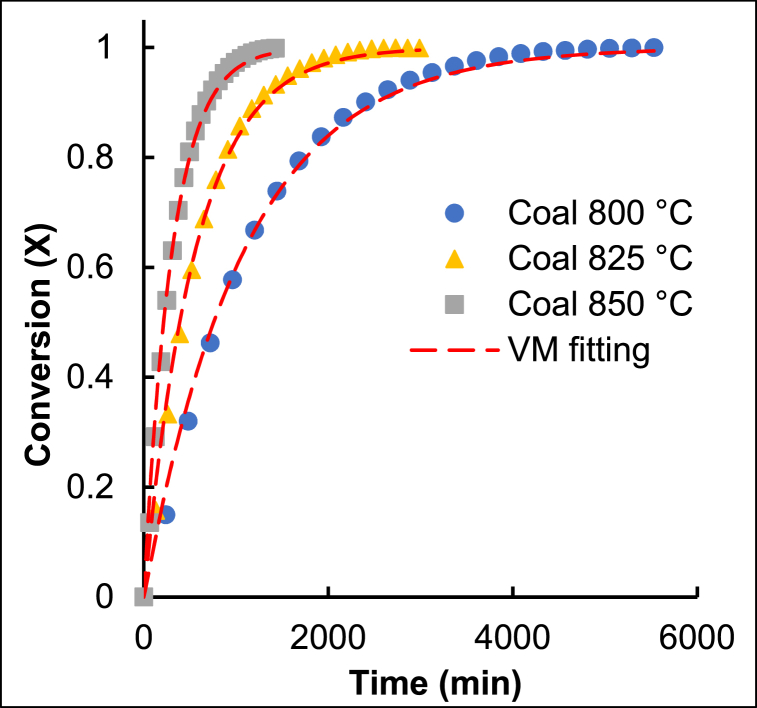
The experimental gasification conversion of coal char compared to the estimated VRM conversions.

The VRM estimated the gasification conversion of the produced chars and was compared to their experimental conversions at various temperatures as demonstrated in Fig. 3.

The remainder of the modelled conversion results compared to the experimental gasification conversion graphs can be found in the Supporting Information. The VRM time factors and the quality of fit for each char type at all three temperatures are summarized in Table 1.

**Table 1:** Gasification reactivity parameters.

Char	Gasification temperature (°C)	t_f VRM_ (min^−1^)	QOF_VRM_ (%)
**Coal fines**	800	9.2E-04	97.9
	825	1.8E-03	97.9
	850	3.2E-03	97.7
**10 % LDPE**	800	1.0E-03	98.8
	825	1.8E-03	97.1
	850	3.5E-03	98.0
**25 % LDPE**	800	1.2E-03	98.6
	825	2.2E-03	98.4
	850	3.9E-03	98.7
**50 % LDPE**	800	1.3E-03	99.1
	825	2.4E-03	98.5
	850	5.0E-03	97.9
**10 % PP**	800	9.9E-04	98.3
	825	1.8E-03	98.1
	850	3.7E-03	98.2
**25 % PP**	800	1.2E-03	99.1
	825	2.0E-03	99.6
	850	4.2E-03	99.4
**50 % PP**	800	1.3E-03	98.8
	825	2.4E-03	99.1
	850	4.5E-03	98.5

The VRM obtained a QOF >97 %. Zhang et al. [25] also reported that the VRM and RPM adequately described the gasification reactivity of polyethylene and semi-coke derived chars.

Therefore, the VRM time factor parameter accurately correlates with the changes in reactivity and consistently increased with an increase in temperature, similar to the results of previously reported results for South African bituminous coals [30,32]. When comparing the time factor of the chars at the same gasification temperature, it increased with an increase in plastic content. At the same plastic content, chars produced from PP and LDPE containing extrudates had similar time factor values, indicating that there is no significant difference in the gasification reactivity when comparing the addition of different plastic types.

The prominent increase in reactivity obtained with an increase in plastic content is somewhat unexpected considering that Marais et al. [10] previously reported that the pyrolysis char yield can overwhelmingly be attributed to the coal fines since the plastics consist almost exclusively of carbon and hydrogen (C and H), thereby yielding high quantities of volatile matter during thermal treatment. Furthermore, the reported carbon and hydrogen content of the chars were similar, and the char yield followed the additive model and did not indicate interaction between the plastics and coal.

The kinetic parameters were determined using the VRM for each char sample. The respective activation energies, pre-exponential factors, and average quality of fit (for all three reaction temperatures) are presented in Table 2.

**Table 2:** Gasification kinetic parameters.

Char	R^2^	E_a_ (kJ.mol^−1^)	k'_s0_ (min^−1^)
**Coal Fines**	0.9997	254	2.0.E+09
**10 % LDPE**	0.9980	248	1.2.E+09
**25 % LDPE**	0.9995	241	6.2.E+08
**50 % LDPE**	0.9971	267	1.3.E+10
**10 % PP**	0.9939	265	8.0.E+09
**25 % PP**	0.9807	241	6.7.E+08
**50 % PP**	0.9998	249	1.8.E+09

The VRM estimated activation energies ranged between 241 and 267 kJ/mol for all the derived chars. These activation energies are slightly higher than the activation energy values of previously reported Highveld coal char which were between 163 and 243 kJ/mol [[42], [43], [44]]. Since all the chars have relatively similar activation energies, the changes in activation energy cannot be the only reason for the sequential increase in reactivity observed with an increase in plastic content. Zhang et al. [25] found that the addition of plastic to semi-coke did result in changes in activation energy, however, the lower activation energies reported did not necessarily correspond to the fastest reactivities obtained. Therefore, the study also concluded that changes in activation energy is not solely responsible for the observed change in reactivity [25].

**Fig. 4:**
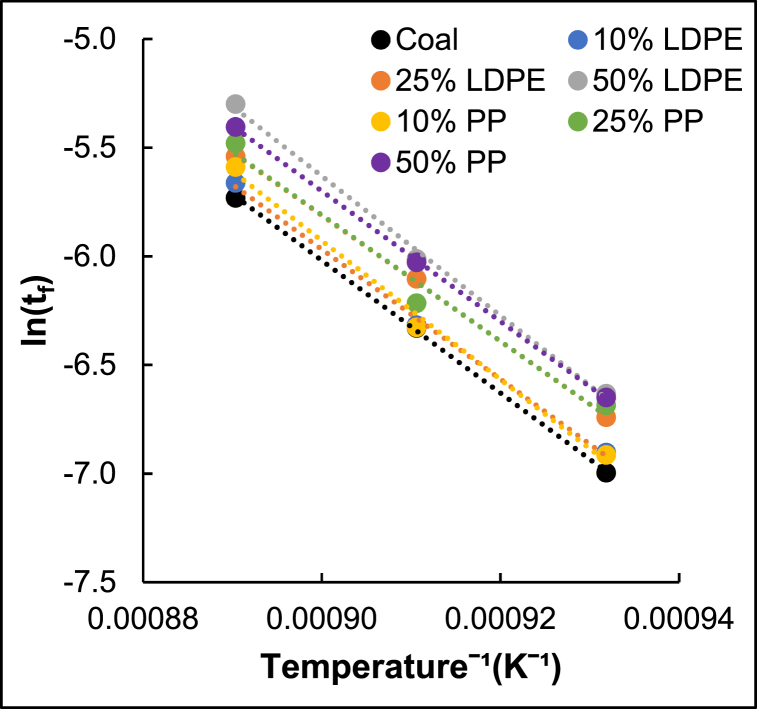
Arrhenius plot for charred samples.

Arrhenius plots were derived from the VRM time factors obtained at all three gasification temperatures. The natural logarithm of the time factors was plotted against T^−1^ for all of the samples as shown in Fig. 4.

Since there is no change in slope for each sample, all the reactions occurred within the same regime. If the regime shifted to the pore diffusion-controlled regime, the activation energy at higher temperatures would have decreased significantly and induced a change in slope. Additionally, the gasification reaction was conducted below 1000 °C (generally low temperatures for gasification) and no shift in slope was observed as shown in Fig. 4, therefore, the chemically controlled regime accurately describes the experimental conditions [45]. The almost parallel lines should indicate similar activation energies [46], thus, changes in activation energy cannot be the reason for the observed increase in reactivity as the plastic content increases (see Fig. 1). To better understand the difference in gasification reactivities observed from the different chars, the changes in evolution of the chars during pyrolysis were further investigated.

### 3.2 Physical-structural properties

#### 3.2.1 SEM

**Fig. 5:**
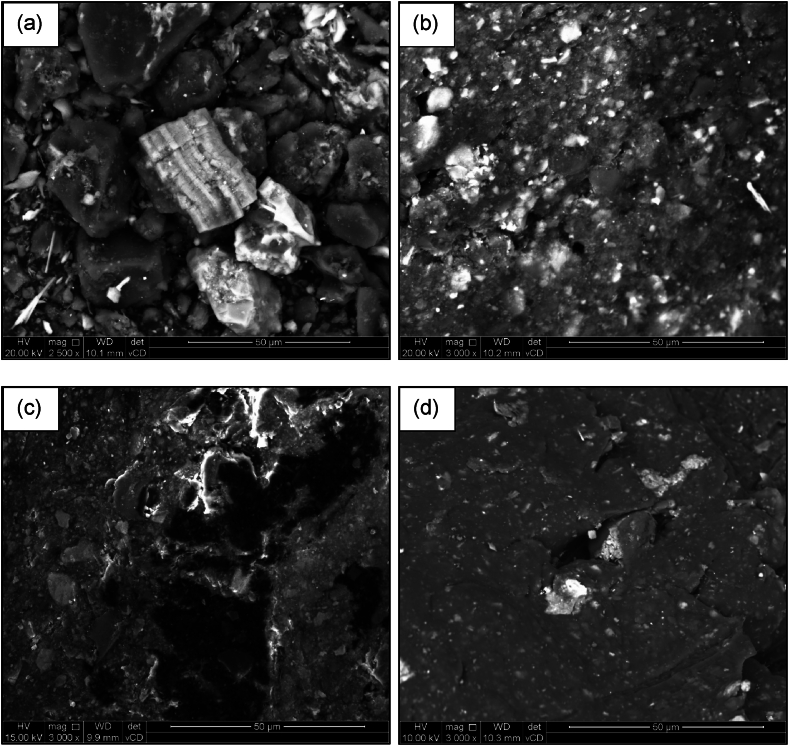
SEM micrographs of the (a) coal fines and LDPE containing extrudates (b) 10 % LDPE, (c) 25 % LDPE, and (d) 50 % LDPE (scale bar 50 μm).

To qualitatively understand the characteristics of the produced chars, the raw coal fines and extrudates were first examined using SEM as shown in Fig. 5.

It can be observed that as soon as plastic is added, the surface is smoother, and the coal particles are surrounded by plastic. The coal particles are evenly spread out indicating that adequate mixing occurred during extrusion. Additionally, as the plastic content increased the spacing between the coal particles increased and the smooth surface of the plastic became more prominent. Zhang et al. [25] observed the evolution of the char during pyrolysis of polyethylene and semi-coke. The SEM images indicated that the coal was still imbedded in the molten plastic until around 500 °C, whereafter the plastic is fully degraded. The same degradation was found in a previous study [9,10]. Since all the plastic has already degraded for the chars produced at the lowest pyrolysis temperature (520 °C), no distinct differences between the char derived from the coal fines and the chars produced from the extrudates were observed through the SEM analyses.

#### 3.2.2 Char form analysis

**Fig. 6:**
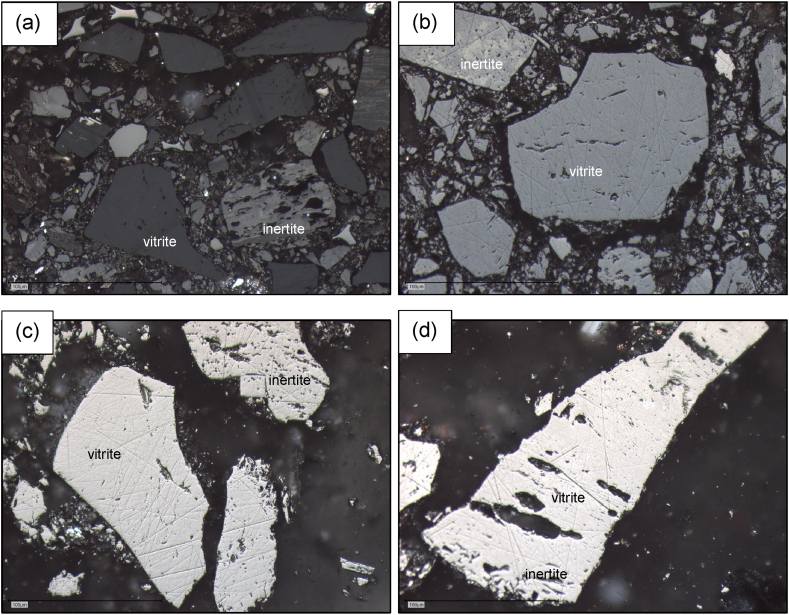
Photomicrographs of pyrolysis at different temperatures (a) raw coal fines, (b) pyrolysis at 520 °C, (c) pyrolysis at 720 °C, and (d) pyrolysis at 920 °C (scale bar 100 μm).

During pyrolysis, the devolatilization of the reactive macerals coincides with the morphological and molecular structural changes of the reactive carbon particles to produce char [14]. The photomicrographs in Fig. 6 shows the evolution of the coal fine chars without any plastic addition as the pyrolysis temperature is increased.

The coal fines (Fig. 6(a)) contain vitrite and inertite and combinations thereof, typical of South African medium rank C bituminous coal fines. As the coal fines underwent pyrolysis, the colour of the vitrite and inertite lightened with an increase in temperature and the release of volatile components [14]. At 520 °C, the vitrite is slightly darker in shade than the inertite (Fig. 6(b)), and all particles show signs of early devolatilization. At 720 °C, the vitrite and inertite particles having the shade of light grey to white are classified as chars. The shade of the vitrite and inertite lightens further at 920 °C indicating that the char underwent further evolution between 720 and 920 °C.

**Fig. 7:**
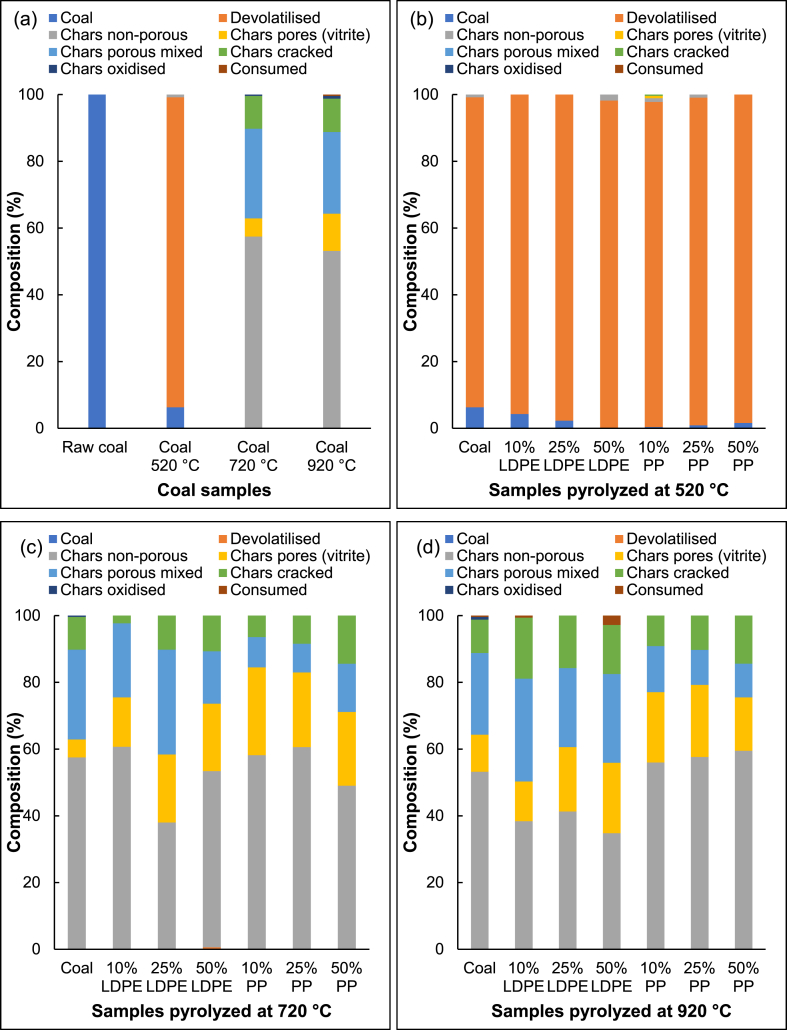
Classification and quantification of the carbon forms present in (a) the coal sample pyrolyzed at different temperatures, (b) samples pyrolyzed at 520 °C (c) samples pyrolyzed at 720 °C and (d) samples pyrolyzed at 920 °C.

The chars derived from the plastic and coal extrudates underwent comparable changes in colour as the pyrolysis temperature increased. To gain insight into the petrographically observable changes, the carbon forms were classified and quantified (Fig. 7).

Fig. 7(a) confirms the progression observed in Fig. 6. The coal sample forms mainly devolatilized coal at 520 °C and consists mainly of chars at 720 °C and 920 °C. The pyrolyzed extrudates showed the same broad char category development. However, differences were observed when evaluating the quantity of material exhibiting pores and cracks. At 520 °C, the samples mostly consist of devolatilized coal, however, the largest portion of the particles has yet to show any signs of pore or crack formation. A small portion of pyrolyzed sample produced from the coal fines is yet to devolatilize (6.3 %), this portion decreases with an increase in LDPE content. The 50 % LDPE containing extrudates pyrolyzed at 520 °C had no observed non-heated particles or remnant coal present. All the pyrolyzed samples that contained PP showed little coal microlithotype particles (<1.6 %) with most particles having been devolatilized. This indicates that addition of plastic accelerated devolatilization at 520 °C.

Furthermore, at 520 °C, the amount of devolatilized coal particles with crack formation derived from the pyrolyzed coal fines (14.5 %) is slightly higher than that produced from the extrudates derived from a 10 % plastic content (LDPE – 10.6 % and PP – 12.3 %). The amount of devolatilized material containing cracks was higher at 25 % and 50 % plastic addition, where 50 % PP contained the most cracked devolatilized coal (17.3 %). Cracking can be attributed to an increase in mechanical or thermal stress [43]. Since these samples were extruded before pyrolysis, cracking due to mechanical stress is possible. However, the 10 % plastic containing samples had to overcome the highest resistance during hot extrusion, since there was only limited molten plastic to ease the flow of material through the extruder. Therefore, if mechanical stress was the main contributor to the crack formation, the 10 % plastic containing extrudates would have exhibited the highest amount of cracking. Since the cracks increased with increasing plastic content, it can be deduced that thermal stress during pyrolysis is possibly the main cause of crack formation. Due to most of the plastics being degraded in a short temperature range [9], the increased volume of volatile matter trying to escape in a short timespan can cause increased stress on the remaining char particles, which could explain the increase in the observed cracking with an increase in plastic content. Furthermore, as the plastic content increased, the amount of devolatilized coal containing pores increased, with the char derived from the 50 % containing plastic extrudates producing the most porous particles observed (LDPE – 20.0 % and PP – 16.4 %). Wu et al. [17] made similar observations when pyrolyzing polyethylene with coal at 550 °C and attributed the more prominent pore formation to the rapid release of volatiles from the polyethylene.

At 720 °C all the samples have converted to chars (as indicated by the white colouration). Once again, the amount of cracking increased with increasing plastic content, and 50 % plastic addition resulted in the most chars forming pores or cracks. The char produced from the coal fines show slight signs of oxidation, which can most likely be attributed to the higher oxygen content present within the coal fines compared to the other plastic-containing samples [9]. A similar slight oxidation effect can also be observed for the char derived from the coal fines produced at 920 °C.

At 920 °C the chars derived from the LDPE containing extrudates produced more signs of cracks and pores than the chars derived from the coal fines and the chars from the PP containing extrudates. The chars derived from the 10 % and 50 % LDPE containing extrudates even contained slight traces of consumed char, once again suggesting that the addition of LDPE accelerated the char formation. Chars derived from PP-containing extrudates showed similar amounts of non-porous char than the char produced from the coal fines, however, the amount of cracking increased with an increase in plastic content. Particles containing cracks have an increased surface area [43], increasing potential active reaction sites which could contribute to the measured increase in gasification rate with an increase in plastic content.

#### 3.2.3 XRD

The relative mineral phase composition of the char samples can be found in the Supporting Information. The mineral phase composition of the chars produced from plastics and coal did not show significant deviance to what was observed in chars produced from raw coal. This was expected since both LDPE and PP have an almost insignificant ash fraction (<2 %) compared to the 29.6 % ash yield fraction obtained for the raw coal [9,10]. The structural parameters of the chars using carbon crystallite analysis were calculated (Table 3).

**Table 3:** Structural parameters of the demineralized chars produced from the coal fines, LDPE containing extrudates, and PP containing extrudates.

Sample	Pyrolysis temperature (°C)	d_002_ (Å)	L_c_ (Å)	L_a_ (Å)	N_ave_
**Coal fines**	0	3.45	9.72	49.68	3.82
**Coal fines**	520	3.51	11.49	23.90	4.27
**10 % LDPE**	520	3.53	11.12	36.36	4.15
**25 % LDPE**	520	3.53	10.59	34.19	4.00
**50 % LDPE**	520	3.52	11.05	36.17	4.14
**10 % PP**	520	3.53	10.91	22.00	4.09
**25 % PP**	520	3.54	11.11	33.65	4.14
**50 % PP**	520	3.52	10.74	33.24	4.05
**Coal fines**	720	3.62	11.29	29.02	4.12
**10 % LDPE**	720	3.58	10.49	40.90	3.93
**25 % LDPE**	720	3.58	10.54	30.89	3.95
**50 % LDPE**	720	3.55	10.21	39.41	3.88
**10 % PP**	720	3.56	10.37	31.57	3.91
**25 % PP**	720	3.59	10.10	39.36	3.81
**50 % PP**	720	3.58	10.34	37.97	3.89
**Coal fines**	920	3.59	10.75	34.16	4.00
**10 % LDPE**	920	3.58	10.74	46.56	4.00
**25 % LDPE**	920	3.62	11.40	37.22	4.15
**50 % LDPE**	920	3.58	10.69	41.03	3.98
**10 % PP**	920	3.58	10.99	37.00	4.07
**25 % PP**	920	3.58	11.04	38.92	4.08
**50 % PP**	920	3.58	11.03	50.79	4.08

It can be observed that the interlayer spacing (d_002_), crystallite height (L_c_), the stacking number of aromatic layers (N_ave_) of all the chars generally increased when compared to the raw coal sample, whereas crystallite diameter (L_a_) of the chars was lower than that of the raw coal fines. These structural changes are due to the carbon crystallites’ exposure to high temperatures which cause the disordered carbon present in the raw coal to become more ordered and better orientated in the produced chars [36].

When comparing the chars produced at different temperature, the interlayer spacing increased with an increase in temperature and at 720 and 920 °C the spacing was relatively similar. The carbon crystallites are more intensely annealed at higher temperatures which increases the interlayer spacing as the disordered carbons of the char are becoming more ordered and orientated [36]. Zhang et al. [18] noted a slight decrease in interlayer spacing when increasing the HDPE content which was co-pyrolyzed with low-rank Chinese coal, however, in the case of this study the change in interlayer spacing with the addition of plastic was found to be insignificant when compared to the effect of temperature.

Additionally, the crystallite height and stacking number of the chars produced from the coal decreased with an increase in temperature; this is in line with previous findings of crystallite condensation where the carbon crystallite size increases in diameter but decreases in height [36]. Yet, the chars produced from the coal and plastic containing extrudates did not behave the same. The char stacking number and crystallite height of the chars produced from the plastic and coal extrudates decreased more significantly between 520 and 720 °C than was the case for the chars derived from the coal fines; but, instead of decreasing further with increasing temperature, as was the case for the stacking number and crystallite height of the char derived from the coal fines, these values increased at 920 °C. Therefore, the crystallite height and stacking values calculated for the chars produced from the extrudates containing plastic at 720 °C had lower calculated values than for any of the other chars, even the chars produced from the coal fines at 920 °C.

The crystallite diameter was also found to increase with an increase in temperature for the chars produced at 920 °C, having the largest crystallite diameter for the chars and closest to that of the raw coal fines. Generally, the chars produced from the extrudates had a larger crystallite diameter than for the chars produced from coal at the same pyrolysis temperature. Furthermore, the chars produced from the extrudates containing PP seems to have a general increase in crystallite diameter as the PP content within the extrudates increased. The char produced from the LDPE containing extrudates have a clear increase in crystallite diameter compared to char produced from the coal at the same pyrolysis temperature, however, there is no gradual increase as the LDPE content in the extrudates further increased. The increase in crystallite diameter indicates a more ordered char with higher degrees of graphitization [32]. This increase in order due to the addition of plastics to coal during pyrolysis was also reported by other studies [18,25].

At 920 °C, the crystallite height and stacking number of the chars produced from the extrudates containing PP are higher than that of the char derived from the coal fines, indicating an increase in skeletal density, order, and degree of graphitization when PP is added to the original sample [18]. It has been theorized that the additional graphitization could be a result of free radicals from the plastic that are absorbed onto the char during pyrolysis, thereby enhancing condensation of aromatic rings [18]. Therefore, comparing the chars produced from the extrudates to the chars produced from the coal at 920 °C, the increase in crystallite diameter found, together with the increase in crystallite height for PP containing extrudates, is not expected to increase the gasification reaction rate, in contrast, it should decrease it since it is known that the growth of crystallite size is associated with a reduction of carbon active sites which halters the gasification rate [47]. Thus, the addition of plastic to coal does not change the carbon crystallite structure in such a way as to promote gasification propensity. However, the reactivity increased with an increase in plastic content, indicating that other changes also occurred to the structure of the char when plastic was added, which positively affected the gasification rate.

#### 3.2.4 CO_2_- and N_2_ LPGA results

During gasification, char-CO_2_ reactions mostly occur via the surface area provided by the mesopores and macropores [48], however, these mesopores develop from micropores in the early stages of char conversion [49]. Therefore, understanding the differences in micropore properties can help to better understand the differences in gasification reaction rates. The micropore properties of the extrudates and derived chars were evaluated using CO_2_ adsorption and are presented in Table 4.

**Table 4:** CO_2_ adsorption micropore properties of the extrudates and derived chars.

Method	Pyrolysis temperature (°C)	Micropore surface area (m^2^/g)	Surface area (m^2^/g)	Maximum pore volume (cm³/g)	Pore width (Å)	Porosity (%)
		D-R	BET	HK	HK	
**Coal**	–	91	72	2.9E-02	3.79	5.41
**10 % LDPE**	–	51	40	1.5E-02	3.92	2.61
**25 % LDPE**	–	47	37	1.4E-02	3.92	2.38
**50 % LDPE**	–	35	28	1.0E-02	3.94	1.75
**10 % PP**	–	57	45	1.7E-02	3.90	3.22
**25 % PP**	–	50	39	1.5E-02	3.93	2.44
**50 % PP**	–	30	24	8.5E-03	3.98	1.36
**Coal**	520	117	90	3.7E-02	3.77	6.84
**10 % LDPE**	520	123	92	4.0E-02	3.67	7.82
**25 % LDPE**	520	115	86	3.8E-02	3.69	7.25
**50 % LDPE**	520	121	91	4.0E-02	3.70	7.63
**10 % PP**	520	124	93	4.0E-02	3.72	7.64
**25 % PP**	520	122	92	4.0E-02	3.70	7.64
**50 % PP**	520	128	96	4.2E-02	3.68	8.11
**Coal**	720	173	128	5.9E-02	3.51	12.36
**10 % LDPE**	720	170	125	5.9E-02	3.45	12.36
**25 % LDPE**	720	172	126	5.9E-02	3.45	12.50
**50 % LDPE**	720	172	126	6.0E-02	3.44	12.56
**10 % PP**	720	176	129	6.0E-02	3.48	12.59
**25 % PP**	720	176	129	6.1E-02	3.46	12.76
**50 % PP**	720	172	127	6.0E-02	3.46	12.55
**Coal**	920	162	126	5.4E-02	3.67	10.55
**10 % LDPE**	920	177	135	6.0E-02	3.61	12.03
**25 % LDPE**	920	178	137	6.2E-02	3.54	12.84
**50 % LDPE**	920	181	138	6.3E-02	3.53	12.94
**10 % PP**	920	180	135	5.9E-02	3.68	11.47
**25 % PP**	920	185	137	6.0E-02	3.69	11.65
**50 % PP**	920	179	134	5.9E-02	3.68	11.38

Before pyrolysis, the extrudates and coal fines have the lowest micropore surface areas, BET surface areas, maximum pore volumes and porosities when compared to their derived chars. As the plastic content in the extrudates is increased, the amount of micropores decreased, most likely being filled by non-permeable melted plastic during hot extrusion. Furthermore, the extrudates and coal fines have a larger pore width than for their respective derived chars, due to the formation of smaller new pores formed at high temperatures.

When comparing the properties of the micropores from the chars produced at different temperatures, the chars produced at 520 °C had the lowest surface area, maximum pore volume, and porosity. This is due to the release of volatile matter and reactions occurring to the carbon structure leaving more porous chars at higher temperatures [50]. Generally, the difference in properties observed for the micropores present in the chars produced at 520 °C when compared to those produced at 720 °C is much larger than the difference observed between the chars produced at 720 and 920 °C since less volatiles are released at higher temperatures [10,27].

At 720 °C, the micropores present in the chars produced from the coal fines are almost identical to those produced from the extrudates. However, the chars produced from the coal fines behave slightly differently when compared to the chars derived from the extrudates when increasing the temperature from 720 to 920 °C. At 920 °C, the micropore surface area, maximum pore volume, and porosity of the chars produced from the coal fines is lower than it is at 720 °C. This is most likely due to the micropores being further developed and coalescing into mesopores, without substantial formation of new micropores to replace them. Since micropores have a small size range, once the pore starts to grow it is a relatively fast transition to a mesopore [49].

However, the extrudates containing LDPE produce chars that exhibit a slight increase in micropore surface area, maximum pore volume, and porosity from 720 to 920 °C, implying that the formation of new micropores was more than sufficient to replace the micropores that grew into mesopores. Furthermore, the CO_2_ adsorption properties of the chars derived from the LDPE containing extrudates increased with an increase in LDPE content, indicating that an increase in LDPE content actively improves micropore formation between 720 and 920 °C.

The extrudates containing PP produced chars at 920 °C that still had increased micropore surface area, maximum pore volume, and porosity when comparing it to the chars produced from the coal fines, yet all of these properties did not increase when compared to the chars produced at 720 °C. Furthermore, the increase in PP content within the extrudates did not result in a continuous increase in the previously mentioned properties as was observed with the extrudates containing LDPE. Therefore, LDPE appears to be more efficient at producing micropores at 920 °C even though the addition of PP still clearly improves the micropore formation of the produced chars. The improvement in pore formation was also reported by Sahu et al. [26], who found that the addition of HDPE to high ash Indian coal produced a char with a larger surface area and CO_2_ adsorption capacity than that of coal alone.

The pyrolysis of both LDPE and PP containing extrudates improved upon the micropore formation of the chars produced at 920 °C, which can definitively be a contributor to the increased gasification reactivity [49] observed in Fig. 1. The N_2_ adsorption pore properties are presented in Table 5.

**Table 5:** N_2_ adsorption pore properties and skeletal density of the extrudates and derived chars.

Method	Temperature (°C)	BET surface area (m^2^/g)	Adsorption pore diameter (Å)	Skeletal density (g/cm³)	Adsorption volume (cm³/g)	Adsorption Total Porosity (cm³/cm³)
		BET	BJH		BJH	
**Coal**	–	6.74	43.92	1.65	2.13E-02	3.52E-02
**10 % LDPE**	–	0.06	558.36	1.44	0.05E-02	0.08E-02
**25 % LDPE**	–	0.13	74.13	1.36	0.09E-02	0.12E-02
**50 % LDPE**	–	0.11	70.11	1.19	0.07E-02	0.09E-02
**10 % PP**	–	0.07	101.87	1.51	0.07E-02	0.10E-02
**25 % PP**	–	0.05	112.67	1.35	0.06E-02	0.07E-02
**50 % PP**	–	0.09	204.70	1.17	0.16E-02	0.18E-02
**Coal**	520	3.82	58.32	1.72	1.18E-02	2.02E-02
**10 % LDPE**	520	13.82	51.86	1.77	2.99E-02	5.28E-02
**25 % LDPE**	520	9.80	84.44	1.75	2.59E-02	4.52E-02
**50 % LDPE**	520	9.73	88.54	1.77	2.80E-02	4.94E-02
**10 % PP**	520	11.14	61.52	1.75	2.51E-02	4.38E-02
**25 % PP**	520	8.83	75.34	1.75	2.17E-02	3.80E-02
**50 % PP**	520	14.20	58.05	1.77	3.01E-02	5.34E-02
**Coal**	720	4.86	447.41	1.93	1.18E-02	2.28E-02
**10 % LDPE**	720	71.11	23.07	1.93	6.04E-02	11.67E-02
**25 % LDPE**	720	68.45	28.32	1.98	4.85E-02	9.58E-02
**50 % LDPE**	720	45.62	107.57	1.99	3.17E-02	6.30E-02
**10 % PP**	720	72.68	20.83	1.98	7.08E-02	14.05E-02
**25 % PP**	720	42.25	123.06	1.97	2.66E-02	5.24E-02
**50 % PP**	720	42.63	119.70	1.96	2.72E-02	5.32E-02
**Coal**	920	3.70	435.88	2.08	1.18E-02	2.45E-02
**10 % LDPE**	920	7.36	200.97	2.19	2.94E-02	6.44E-02
**25 % LDPE**	920	14.95	50.80	2.20	3.18E-02	6.99E-02
**50 % LDPE**	920	16.63	45.66	2.22	3.15E-02	7.01E-02
**10 % PP**	920	9.21	132.68	2.14	2.63E-02	5.63E-02
**25 % PP**	920	8.90	205.21	2.15	3.55E-02	7.62E-02
**50 % PP**	920	11.35	79.58	2.01	2.39E-02	4.79E-02

Table 5 shows that the extrudates containing either plastic type has a very low surface area, adsorption volume, and total porosity. This is due to the melted plastics filling most of the existing cracks and pores of the coal fines, and plastics are by nature non-porous. In Fig. 2(b–d) the plastic can be seen covering the fine coal particles. Since most of the pores are filled with plastic during extrusion, there is no obvious trend when observing the average pore diameter of the few remaining open pores. Furthermore, the skeletal density of the extrudates decreases linearly as the plastic content increases, since both LDPE and PP have a lower skeletal density than that of the coal fines [9]. The chars derived from the extrudates all have higher surface areas, skeletal densities, adsorption volumes, and porosities when compared to the properties of the chars produced from the coal fines pyrolyzed at the same temperature.

The increase in skeletal density is most likely a further indication of a more ordered carbon structure. This should also be a sign of decreasing reactivity since a more ordered carbon structure containing larger carbon crystallites is evident and thereby, limiting the amount of available reaction active sites [47]. However, the reactivity of the chars still increase even with an increase in skeletal density, which can be attributed to the increase in other structural properties like surface area when comparing the coal char to the chars derived from the extrudates.

Zhou et al. [51] reported that PP almost completely degrades (98 %) in a narrow temperature range between 399 and 507 °C and that LDPE similarly degrades (99 %) between 426 and 526 °C. A previous study found similar results with a final degradation temperatures of 505 °C for PP and 520 °C for LDPE [9]. Therefore, when pyrolyzed at 520 °C, both plastics have largely degraded producing large amounts of volatile matter (dependent on the concentration within the extrudate) which contributes to the formation of pores. The pores produced from the extrudates generally have more than double the surface area, adsorption volume, and porosity when compared to the those of the chars produced from the coal fines, whereas the average pore diameter is in a similar range (between 50 and 90 Å). This indicates that the chars produced from the extrudates are more meso-porous.

At 720 °C, the difference in properties of the pores produced by the extrudates when compared to those of the chars produced from the coal fines become even more pronounced, especially the difference in surface area. The lowest surface area recorded for the chars produced from the extrudates at 720 °C is 42.25 m^2^/g (25 % PP); this is almost 10 times the surface area of the chars produced from the coal fines (4.86 m^2^/g). The average pore diameter of the char derived from the coal fines (447.41 Å) is almost four times larger than the average diameter of the nearest char produced from the extrudates. This can be an indication that the existing pores of the coal fines grew due to thermal stress [49] without developing new pores, whereas the chars produced from the extrudates developed new pores and this is why the average pore diameter is much lower. The continuous development of new pores from the extrudates is confirmed by the increase in micropore volume and porosity (Table 4).

**Fig. 8:**
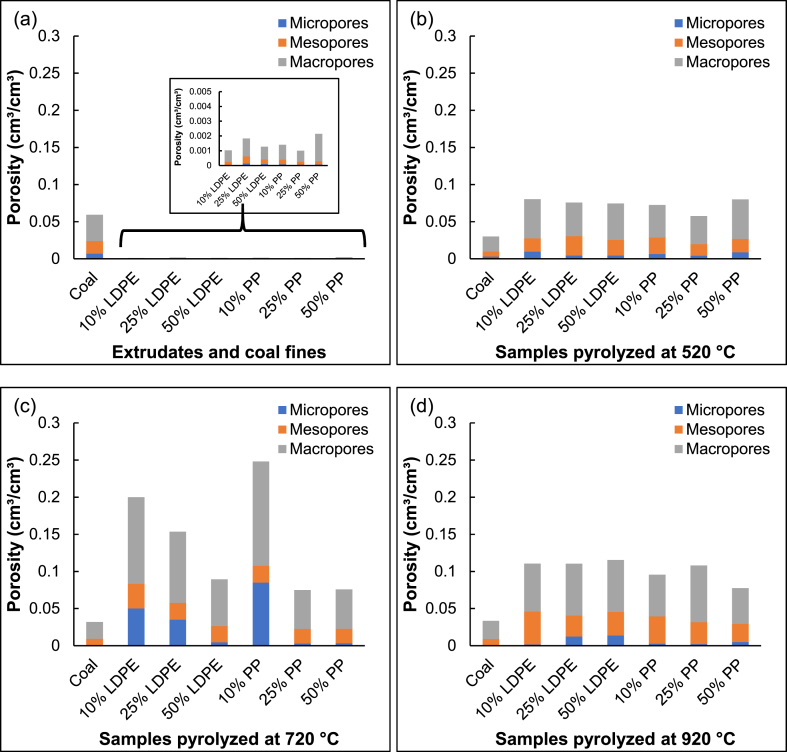
The overall porosity of the (a) coal fines and extrudates, and the produced chars pyrolyzed at (b) 520 °C, (c) 720 °C, and (d) 920 °C.

Fig. 8 shows the pore size distribution of the N_2_ adsorption porosity, helping to evaluate the contributions of the micro-, meso-, and macropores to the overall porosity of the samples and the produced chars.

It can be seen from Fig. 8 that the largest contributor to the overall porosity are macropores, since the increased diameter increases the pore volume. The mesopores generally contribute the second most, and the small diameter of the micropores contributing the least. However, at 720 °C, the micropores significantly contribute to the porosity of the extrudates containing a lower plastic content (10 % LDPE, 25 % LDPE, and 10 % PP). This increased microporosity could be because the extrudates tend to maintain their structures best at lower plastic concentrations during pyrolysis (Fig. 1) which possibly entraps the volatile matter within the structure. The entrapment of the volatile matter within the structure exposes the char to the volatile matter for longer periods which can lead to increased interactions and the possible production of micropores. Another possibility is that there is more coal relative to the plastic content, which results in more intense contact when the plastic is volatilized. These micropores are consumed by the evolution of larger pores during pore coalescence when pyrolyzed at 920 °C, however, the porosity of the chars produced from the extrudates is still considerably higher than that for the chars derived from the coal fines. The increase in porosity provides more surface area and increase the availability of reaction active sites. Most of the gasification reactions occur based on the surface area of the mesopores and macopores [48].

When the chars are pyrolyzed at 920 °C, the surface area is greatly reduced when compared to chars produced at 720 °C. This can be attributed to the collapse of the carbon skeleton of coal, which is known to collapse at higher temperatures thereby reducing the available surface area [52]. Therefore, from 720 to 920 °C the shift most likely occurs where pore growth is no longer dominant and pore coalescence is more prominent.

**Fig. 9:**
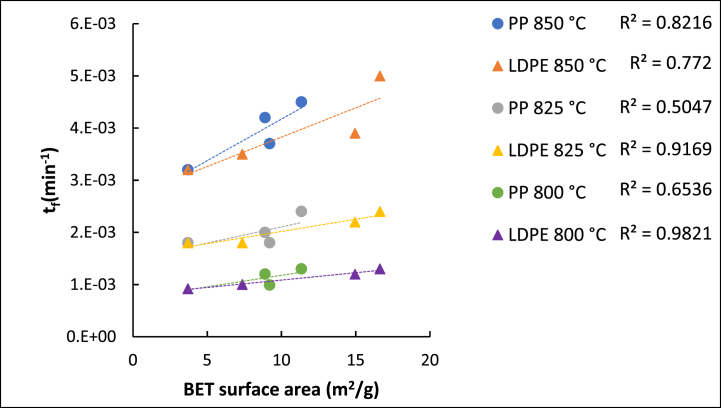
BET Surface area compared to the volumetric time factor for the gasification of the chars pyrolyzed at 920 °C.

Even though the surface area of the chars produced from the extrudates decreases from 720 to 920 °C, the surface area, adsorption volume, and porosity of these chars are still clearly higher than the char produced from the coal. The increase in these properties observed when comparing the coal char to the chars of the extrudates produced at 920 °C provides a larger reaction zone for the CO_2_ gasification reaction to occur, resulting in higher reactivities. This is demonstrated by plotting the time factor of the VRM against the BET surface area (Fig. 9).

A positive linear correlation was found for both the chars derived from LDPE and PP extrudates, however, the chars derived from the LDPE obtained better R^2^ values, indicating a stronger linear correlation. Even though the surface area of the chars derived from PP extrudates is much higher than that of the coal, the 25 % PP derived char had a slightly lower surface area than the 10 % PP derived char, whereas the chars derived from the LDPE extrudates had a continuous increase in surface area with increase in LDPE content (Table 5). Since the surface area can be positively correlated to the VRM time factor, it can directly be correlated to the increase in reactivity. Additionally, most of the gasification reactions occur based on the surface area of the mesopores and macopores [48], of which the chars produced from the extrudates had increased mesopore and macropore porosity when comparing the chars produced from the coal at 920 °C (Fig. 9). Therefore, the increase in meso- and macropores can be correlated to the increase in measured gasification reactivity observed with increasing plastic content.

## 4 Concluding remarks

The pyrolysis chars derived from extrudates containing plastic and coal showed an increase in gasification reactivity with an increase in plastic content for both LDPE and PP containing extrudates. At similar plastic content, the reactivities of the chars derived from LDPE and PP were similar, indicating that there was no significant difference in reactivity when different plastics were used.

With increasing pyrolysis temperature, the chars produced from the extrudates containing plastic continuously demonstrated higher degrees of pore and crack formation when compared to the chars produced from the coal fines. Even though the XRD analysis of the carbon crystalline structure indicated that the addition of plastic lead to the production of more ordered chars and increasing crystallite size, the addition of plastic to the raw extrudate also resulted in more abundant pore formation, which lead to a net increase in gasification reactivity. Changes in activation energy was not responsible for the increase in observed reactivity, since all the pulverized chars had similar activation energies ranging between 241 and 268 kJ/mol.

The LPGA results reports that maximum porosity occurred at 720 °C and decreased when the pyrolysis temperature further increased. This indicates that pore coalescence mainly occurs during gasification and that most of the pore growth occurred during the pyrolysis stage. At 920 °C the chars produced from the extrudates demonstrated an increase in meso- and macro-porosity, compared to the char derived from the coal. The volumetric reaction model (VRM) adequately describes the gasification kinetics of the pulverized chars. The VRM time factor showed a positive linear correlation with an increase in surface area, especially with for the LDPE containing chars (R^2^ > 0.7720). Therefore, the reactivity increase observed with an increase in plastic content can be correlated to an increase in surface area.

It can be concluded that pyrolysis of the extrudates containing plastic and coal fines formulated a fuel source that has an increased gasification rate. Therefore, the gasification of plastics and coal fines has the prospect of reducing two major waste streams while potentially increasing gasification rates which could increase production rates for industrial fixed bed gasification. Since thermal fragmentation during pyrolysis was found to destroy the integrity of the chars formed from the extrudates at plastic additions >10 %, further work should be conducted with the addition of suitable binders to minimise the thermal fragmentation propensity of the plastic-containing coal bound extrudates in order to make the extrudates suitable for the fixed bed gasification application.

## CRediT authorship contribution statement

**C. Marais:** Writing – original draft, Visualization, Methodology, Formal analysis, Conceptualization. **J.R. Bunt:** Writing – review & editing, Supervision, Methodology, Conceptualization. **N.T. Leokaoke:** Writing – review & editing, Supervision, Methodology. **H.W.J.P. Neomagus:** Writing – review & editing, Supervision, Methodology. **G.N. Okolo:** Writing – review & editing, Methodology, Formal analysis. **N.J. Wagner:** Writing – review & editing, Methodology, Formal analysis. **J.A. Meyer:** Writing – review & editing, Methodology.

## Declaration of competing interest

The authors declare the following financial interests/personal relationships which may be considered as potential competing interests:Carel Marais reports financial support was provided by 10.13039/501100001321National Research Foundation. If there are other authors, they declare that they have no known competing financial interests or personal relationships that could have appeared to influence the work reported in this paper.
