# Supplementary material for: Structural analysis and gasification reactivity of chars derived from the slow pyrolysis of extruded coal fines and recycled plastic
Source: Heliyon. 2024 Oct 15;10(20):e39391. doi: 10.1016/j.heliyon.2024.e39391 (PMC11513508; doi:10.1016/j.heliyon.2024.e39391)
Supplement: Multimedia component 1 [file mmc1.docx]

**Supporting information: Structural analysis and gasification reactivity of chars derived from the slow pyrolysis of extruded coal fines and recycled plastic**

C. Marais^1^, J.R. Bunt^1,^*, N.T. Leokaoke^1^, H.W.J.P. Neomagus^1^, G.N. Okolo^1^, N.J. Wagner^2^, J.A. Meyer^1^

^1^Centre of Excellence in Carbon-Based Fuels, North-West University, Potchefstroom Campus, Private Bag X6001, Potchefstroom 2520, South Africa.

^2^DSI-NRF CIMERA, University of Johannesburg.

*Corresponding author: E-mail: john.bunt@nwu.ac.za

| **Nomenclature** | | |
| --- | --- | --- |
| **Symbol** | **Description** | **Units** |
| \| $E_{a}$ \| \| --- \| | Activation Energy | *kJ.mol^-1^* |
| \| ${k'}_{s0}$ \| \| --- \| | Lumped pre-exponential factor | *min^-1^* |
| \| $r_{s}$ \| \| --- \| | Intrinsic reaction rate | *m.min^-1^* |
| \| $S_{0}$ \| \| --- \| | Initial Surface area | *m^2^.m^-3^* |
| $t$ | reaction real time | *min* |
| $t_{f}$ | time factor | *min^-1^* |
| \| $QOF$ \| \| --- \| | Quality of fit | *%* |
| $X$ | Conversion | *-* |
| \| $\varepsilon_{0}$ \| \| --- \| | Initial porosity | *-* |
| $\psi$ | Dimensionless structural parameter | *-* |

## **Appendix 1: Random pore model (RPM) experimental method**

The RPM considers the random pore orientation and size distribution^1–3^. This model provides an explanation for the changes in the char’s surface as the reaction proceeds, including pore growth and the formation of overlapping pores which reduces the available surface area^2,3^. The RPM is given by Equation S1^3,4^.

$$\begin{aligned} \frac{dX}{dt}=\frac{r_{s}S_{0}\left( 1-X \right)\sqrt{[1-\psi\ln\left( 1 - X \right) ]}}{(1-\varepsilon_{0})}\#\left( S1 \right) \end{aligned}$$

Where $r_{s}$ is the intrinsic reaction rate, and the dimensionless structural parameter ($\psi$) is described by Equation S2.

$$\begin{aligned} \psi=\frac{4\pi L_{0}\left( 1-\varepsilon_{0} \right)}{{S_{0}}^{2}}\#\left( S2 \right) \end{aligned}$$

$L_{0}$ is the total length per unit volume and $\varepsilon_{0}$, and $S_{0}$ are the initial porosity and surface area, respectively. The RPM gasification conversion is provided by Equation S3.

$$\begin{aligned} X=1-\exp\left[ -t_{f}t\left( 1+\frac{t_{f}t\psi}{4} \right) \right]\#\left( S3 \right) \end{aligned}$$

Where $t_{f}$ is now the time factor when using the RPM and $t$ is still the reaction real time. The structural parameter can be estimated by linear regression using the dimensionless reduced time, $t/t_{0.9}$ (Equation S4)^5^.

$$\begin{aligned} \frac{t}{t_{0.9}}=\frac{\sqrt{1-\psi\ln\left( 1-X \right)}-1}{\sqrt{1-\psi\ln\left( 1-0.9 \right)}-1}\#\left( S4 \right) \end{aligned}$$

## **Appendix 2: Comparison between RPM and VRM**

The char gasification was modelled using both the RPM and VRM. These models provided similar kinetic parameters and quality of fit results as presented in Figure S1, Table S1, and Table S2. When the structural parameter (Ψ_RPM_) is equal to zero (Table S2), the RPM reduces exactly to the VRM. Since the structural parameter is very close to zero, the VRM and RPM provide similar results, and both models deliver satisfactory QOF-values for all of the chars. Therefore, the simpler VRM model was used to further discuss the gasification kinetics of the chars.

Figure S1: The experimental gasification conversion compared to the estimated RPM and VRM conversions of the chars produced from the extrudates containing (a) coal, (b) 10% LDPE, (c) 10% PP, (d) 25% LDPE, (e) 25% PP, (f) 50% LDPE, (g) 50% PP.

Table S1: Gasification kinetic parameters

| **Char** | **Reactivity model** | **R²** | **E_a_ (kJ.mol^-1^)** | **k’_s0_**  **(min^-1^)** |
| --- | --- | --- | --- | --- |
| **Coal Fines** | R_RPM_ | 0.9999 | 253 | 1.6.E+09 |
|  | R_VRM_ | 0.9997 | 254 | 2.0.E+09 |
| **10% LDPE** | R_RPM_ | 0.9856 | 244 | 6.4.E+08 |
|  | R_VRM_ | 0.9980 | 248 | 1.2.E+09 |
| **25% LDPE** | R_RPM_ | 1.0000 | 242 | 6.4.E+08 |
|  | R_VRM_ | 0.9995 | 241 | 6.2.E+08 |
| **50% LDPE** | R_RPM_ | 0.9954 | 252 | 2.1.E+09 |
|  | R_VRM_ | 0.9971 | 267 | 1.3.E+10 |
| **10% PP** | R_RPM_ | 0.9955 | 262 | 4.9.E+09 |
|  | R_VRM_ | 0.9939 | 265 | 8.0.E+09 |
| **25% PP** | R_RPM_ | 0.9878 | 245 | 9.5.E+08 |
|  | R_VRM_ | 0.9807 | 241 | 6.7.E+08 |
| **50% PP** | R_RPM_ | 0.9996 | 253 | 2.3.E+09 |
|  | R_VRM_ | 0.9998 | 249 | 1.8.E+09 |

The reactivity values, reaction times, time factors for the RPM and VRM, the dimensionless structural parameters of the RPM, and the quality of fit for each char type at all three temperatures are summarized in Table S2.

Table S2: Gasification reactivity parameters

| **Char** | **Gasification temperature (°C)** | **t_f RPM_ (min^-1^)** | **Ψ_RPM_** | **QOF_RPM_ (%)** | **t_f VRM_ (min^-1^)** | **QOF_VRM_ (%)** |
| --- | --- | --- | --- | --- | --- | --- |
| **Coal fines** | 800 | 8.0E-04 | 0.47 | 99.0 | 9.2E-04 | 97.9 |
|  | 825 | 1.5E-03 | 0.51 | 99.0 | 1.8E-03 | 97.9 |
|  | 850 | 2.8E-03 | 0.49 | 98.9 | 3.2E-03 | 97.7 |
| **10% LDPE** | 800 | 9.0E-04 | 0.34 | 99.2 | 1.0E-03 | 98.8 |
|  | 825 | 1.5E-03 | 0.77 | 99.1 | 1.8E-03 | 97.1 |
|  | 850 | 3.0E-03 | 0.47 | 99.3 | 3.5E-03 | 98.0 |
| **25% LDPE** | 800 | 1.1E-03 | 0.29 | 99.4 | 1.2E-03 | 98.6 |
|  | 825 | 2.0E-03 | 0.36 | 99.5 | 2.2E-03 | 98.4 |
|  | 850 | 3.6E-03 | 0.26 | 99.6 | 3.9E-03 | 98.7 |
| **50% LDPE** | 800 | 1.2E-03 | 0.18 | 99.5 | 1.3E-03 | 99.1 |
|  | 825 | 2.2E-03 | 0.36 | 99.5 | 2.4E-03 | 98.5 |
|  | 850 | 4.4E-03 | 0.15 | 99.7 | 5.0E-03 | 97.9 |
| **10% PP** | 800 | 9.0E-04 | 0.33 | 99.2 | 9.9E-04 | 98.3 |
|  | 825 | 1.6E-03 | 0.32 | 99.0 | 1.8E-03 | 98.1 |
|  | 850 | 3.3E-03 | 0.28 | 99.3 | 3.7E-03 | 98.2 |
| **25% PP** | 800 | 1.2E-03 | 0.17 | 99.5 | 1.2E-03 | 99.1 |
|  | 825 | 2.0E-03 | 0.06 | 99.6 | 2.0E-03 | 99.6 |
|  | 850 | 4.0E-03 | 0.12 | 99.7 | 4.2E-03 | 99.4 |
| **50% PP** | 800 | 1.2E-03 | 0.38 | 99.7 | 1.3E-03 | 98.8 |
|  | 825 | 2.2E-03 | 0.22 | 99.7 | 2.4E-03 | 99.1 |
|  | 850 | 4.1E-03 | 0.32 | 99.7 | 4.5E-03 | 98.5 |

## **Appendix 3: Mineral phase composition**

The relative mineral phase composition of the raw coal and char samples is provided in Figure S2.

\

Figure S2: Mineral phases of (a) the raw coal compared to the char produced from coal at 520, 720 and 920 °C, (b) chars produced at 520 °C, (c) chars produced at 720 °C and (d) chars produced at 920 °C

Figure S2(a) indicates that the organic carbon is the most abundant in the raw material and in all of its produced chars. The organic carbon content increases when charred due to the release of volatile matter and moisture content. The most prominent mineral phase in the raw coal and the char produced at 520 °C is kaolinite (Al_2_Si_2_O_5_(OH)_4_), however, the chars produced at higher temperatures have almost no kaolinite present. This is due to the dehydroxylation of kaolinite between 450 and 700 °C to produce metakaolinite^6^. Metakoalinite does not appear in the mineral phase XRD analysis due to its amorphous layered structure preventing it from producing crystalline XRD diffraction lines^7,8^. Since there is no metakaolinite shown in the mineral phases, the other minerals appear more abundant resulting in the appearance of quartz being the most prominent mineral phase at higher temperatures.

The mineral phase composition of the chars produced from plastics and coal did not show significant deviance to what was observed in chars produced from raw coal. This was expected since both LDPE and PP have an almost insignificant ash fraction (< 2%) compared to the 29.6% ash yield fraction obtained from the raw coal^9,10^.

## **Supporting information references**

1. Uwaoma RC, Strydom CA, Bunt JR, Matjie RH. Gasification of chars from tetralin liquefaction of < 1.5 g cm−3 carbon‑rich residues derived from waste coal fines in South Africa. *J Therm Anal Calorim*. 2022;147:2353-2367. doi:https://doi.org/10.1007/s10973-021-10609-5 Gasification

2. Roncancio R, Gore JP. CO2 char gasification: A systematic review from 2014 to 2020. *Energy Convers Manag X*. 2021;10(December 2020):100060. doi:10.1016/j.ecmx.2020.100060

3. Bhatia SK, Perlmutter DD. A Rondom Pore Model for Fluid-Solid Reactions: 1. Isothermal , Kinetic Control. *AIChE J*. 1980;26(3):379-386. doi:https://doi.org/10.1002/aic.690260308

4. Meyer NJA, Strydom CA, Bunt JR, Uwaoma RC. Direct Liquefaction of South African Vitrinite- and Inertinite-Rich Coal Fines. *ACS Omega*. 2024. doi:10.1021/acsomega.4c00602

5. Everson RC, Neomagus HWJP, Kaitano R, Falcon R, du Cann VM. Properties of high ash coal-char particles derived from inertinite-rich coal: II. Gasification kinetics with carbon dioxide. *Fuel*. 2008;87(15-16):3403-3408. doi:10.1016/j.fuel.2008.05.019

6. Ptáčeka P, Kubátová D, Havlica J, Brandˇstetr J, Šoukal F, Opravil T. Isothermal kinetic analysis of the thermal decomposition of kaolinite: The thermogravimetric study. *Thermochim Acta J*. 2010;501:24-29. doi:10.1016/j.tca.2009.12.018

7. Wang J, Ishida R, Takarada T. Carbothermal Reactions of Quartz and Kaolinite with Coal Char. *Energy and Fuels*. 2000;14(5):1108-1114. doi:https://doi.org/10.1021/ef000084x

8. Machida S, Katsumata K, Yasumori A. Effects of kaolinite layer expansion and impurities on the solid-state reaction of kaolinite. *RSC Adv*. 2021;11:38473-38477. doi:10.1039/d1ra07762g

9. Marais C, Bunt JR, Leokaoke NT, Uwaoma RC, Neomagus HWJP. Mechanical and Thermal Properties of Extrudates Produced from Discarded Coal Fines and Recycled Plastics as Binders. *Energy and Fuels*. 2023;(37):5905-5916. doi:10.1021/acs.energyfuels.3c00514

10. Marais C, Bunt JR, Leokaoke NT, Coetzer RLJ, Neomagus HWJP. Slow Pyrolysis Products Derived from Extrudates Produced from Discard Coal Fines and Recycled Plastics as Binders. *ACS Omega*. 2024;9:6627-6641. doi:10.1021/acsomega.3c07626
